# Supplementary material for: The Advanced Organ Support (ADVOS) hemodialysis system fulfills its intended purpose: Analysis of data from 282 patients from the Registry on Extracorporeal Multiple Organ Support (EMOS)
Source: PLoS One. 2025 Dec 4;20(12):e0318917. doi: 10.1371/journal.pone.0318917 (PMC12677454; doi:10.1371/journal.pone.0318917)
Supplement: S1 File — (DOCX) [file pone.0318917.s003.docx]

**Supplementary Information**

**Supplementary Table 1**. Baseline characteristics immediately before the 1st ADVOS treatment session in each of the subgroups. Median (IQR) or percentage.

|  | **Acidosis (n = 146)** | | | **No Acidosis (n = 134)** | | | **ACLF 3 (n = 53)** | | |
| --- | --- | --- | --- | --- | --- | --- | --- | --- | --- |
| **Parameters** | Median | IQR 25 | IQR 75 | Median | IQR 25 | IQR 75 | Median | IQR 25 | IQR 75 |
| Age | 57 | 48 | 67 | 59 | 45,5 | 69 | 54,5 | 39,75 | 61 |
| Sex (male, %) | 96 | 66% |  | 81 | 61% |  | 37 | 71% |  |
| Reason for admission (n, %) |  |  |  |  |  |  |  |  |  |
| Emergency | 69 | 48% |  | 57 | 43% |  | 26 | 50% |  |
| Planned admission (e.g. diagnostic, surgery) | 17 | 12% |  | 31 | 23% |  | 4 | 8% |  |
| Bridging to Transplantation | 4 | 3% |  | 3 | 2% |  | 1 | 2% |  |
| Transfer from other hospital | 54 | 37% |  | 42 | 32% |  | 21 | 40% |  |
| Alcohol abuse (n, %) | 61 | 42% |  | 51 | 38% |  | 35 | 67% |  |
| Smoker (n, %) | 31 | 21% |  | 30 | 23% |  | 11 | 21% |  |
| Body height | 175 | 168 | 180 | 173 | 1,67 | 180 | 175 | 168 | 180 |
| Body weight | 80 | 73 | 98 | 75 | 68 | 89 | 80 | 70 | 86 |
| Vasoactive and supportive substances (n, %) | 133 | 91% |  | 97 | 72% |  | 48 | 91% |  |
| Mechanical ventilation (n, %) | 111 | 76% |  | 79 | 59% |  | 31 | 58% |  |
| Acidosis (n, %) | 146 | 100% |  | 0 | 0% |  | 31 | 58% |  |
| Acute-on-chronic-liver failure (ACLF)* |  |  |  |  |  |  |  |  |  |
| pre-existing liver disease | 87 | 60% |  | 79 | 59% |  | 53 | 100% |  |
| CLIF-C-ACLF Score documented | 46 | 32% |  | 41 | 31% |  | 53 | 100% |  |
| CLF-C-ACLF Score | 61 | 56 | 57 | 58 | 46 | 67 | 66 | 59 | 72 |
| no ACLF | 0 | 0% |  | 1 | 1% |  | 0 | 0% |  |
| ACLF grade 1 | 4 | 3% |  | 4 | 3% |  | 0 | 0% |  |
| ACLF grade 2 | 11 | 8% |  | 15 | 11% |  | 0 | 0% |  |
| ACLF grade 3 | 31 | 21% |  | 21 | 16% |  | 53 | 100% |  |
| Glasgow Coma Score | 5 | 3 | 15 | 10 | 3 | 15 | 14 | 5 | 15 |
| Hepatic Encephalopathy Grade | 0 | 0 | 2 | 0 | 0 | 1,75 | 1 | 0 | 2,5 |
| No Encephalopathy | 69 | 47% |  | 51 | 38% |  | 21 | 40% |  |
| Grade 1 | 9 | 6% |  | 9 | 7% |  | 10 | 19% |  |
| Grade 2 | 16 | 11% |  | 8 | 6% |  | 9 | 17% |  |
| Grade 3 | 7 | 5% |  | 9 | 7% |  | 8 | 15% |  |
| Grade 4 | 3 | 2% |  | 3 | 2% |  | 5 | 9% |  |
| SOFA Score | 16 | 13 | 18 | 14 | 12 | 16 | 15 | 13 | 18 |
| SOFA Cardiac | 4 | 4 | 4 | 3 | 1 | 4 | 4 | 2 | 4 |
| SOFA Respiratory | 2 | 2 | 3 | 3 | 2 | 3 | 3 | 1 | 3 |
| SOFA Liver | 2 | 2 | 4 | 4 | 2 | 4 | 4 | 2 | 4 |
| SOFA Kidney | 4 | 2 | 4 | 2 | 0 | 4 | 4 | 3 | 4 |
| SOFA Coagulation | 2 | 0 | 3 | 2 | 1 | 3 | 2 | 0 | 3 |
| SOFA GCS | 4 | 0 | 4 | 2 | 0 | 4 | 1 | 0 | 4 |
| Comorbidities at hospital admission (n, %) |  |  |  |  |  |  |  |  |  |
| Myocardial infarction | 4 | 3% |  | 12 | 9% |  | 1 | 2% |  |
| Congestive heart failure | 26 | 18% |  | 24 | 18% |  | 6 | 12% |  |
| Peripheral vascular disease | 10 | 7% |  | 10 | 8% |  | 3 | 6% |  |
| Cerebrovascular disease | 9 | 6% |  | 6 | 5% |  | 2 | 4% |  |
| Dementia | 1 | 0,7% |  | 0 | 0% |  | 0 | 0% |  |
| Chronic pulmonary disease | 22 | 15% |  | 13 | 10% |  | 2 | 4% |  |
| Rheumatologic disease | 4 | 3% |  | 2 | 2% |  | 0 | 0% |  |
| Peptic ulcer disease | 16 | 11% |  | 14 | 11% |  | 7 | 13% |  |
| Mild liver disease | 19 | 13% |  | 12 | 9% |  | 6 | 12% |  |
| Diabetes without chronic complications (without end organ damage) | 38 | 26% |  | 28 | 21% |  | 12 | 23% |  |
| Diabetes with chronic complications (with end organ damage) | 5 | 3% |  | 3 | 2% |  | 0 | 0% |  |
| Moderate or severe renal (kidney) disease | 26 | 18% |  | 22 | 17% |  | 10 | 19% |  |
| Hemiplegia or paraplegia | 2 | 1% |  | 3 | 2% |  | 0 | 0% |  |
| Malignancy (during last 5 years) | 19 | 13% |  | 20 | 15% |  | 1 | 2% |  |
| Leukemia | 4 | 3% |  | 1 | 1% |  | 0 | 0% |  |
| Lymphoma | 2 | 1% |  | 6 | 5% |  | 0 | 0% |  |
| Moderate or severe liver disease | 69 | 48% |  | 67 | 50% |  | 47 | 90% |  |
| Metastatic solid tumor | 7 | 5% |  | 5 | 4% |  | 1 | 2% |  |
| AIDS / HIV | 2 | 1% |  | 1 | 1% |  | 1 | 2% |  |

*Incomplete data at baseline did not allow to calculate the CLIF-C-ACLF Score in many cases. This may lead to an underestimation of the number of ACLF patients.

**Supplementary Table 2**. ADVOS Treatment settings in each of the subgroups. Median (IQR). The UF rate also includes the volume corresponding to potential glucose, citrate or calcium administration, which account to approximately 70-120 mL/h.

|  | **Acidosis (n = 146)** | | | **No Acidosis (n = 134)** | | | **ACLF 3 (n=53)** | | |
| --- | --- | --- | --- | --- | --- | --- | --- | --- | --- |
| **Treatment settings** | Median | IQR 25 | IQR 75 | Median | IQR 25 | IQR 75 | Median | IQR 25 | IQR 75 |
| Total number of treatment sessions | 495 |  |  | 564 |  |  | 233 |  |  |
| Treatment session/patient | 3 | 1 | 4 | 3 | 2 | 5,75 | 3 | 2 | 6 |
| Median treatment duration (h) | 19 | 12 | 24 | 18 | 7 | 22 | 16 | 9 | 21 |
| Median blood flow (mL/min) | 100 | 100 | 150 | 100 | 100 | 150 | 120 | 100 | 150 |
| Median concentrate flow (mL/min) | 160 | 160 | 160 | 160 | 160 | 315 | 160 | 160 | 300 |
| Median pH value | 8,0 | 7,6 | 9,0 | 7,4 | 7,4 | 7,8 | 7,8 | 7,4 | 8,7 |
| Median Ultrafiltration rate (mL/h) | 100 | 10 | 250 | 152,5 | 70 | 260 | 250 | 79 | 300 |
| Average Ultrafiltration volume (mL) | 2400 | 1119 | 4722 | 3212 | 525 | 5000 | 2844 | 1138 | 5294 |
| Patients with treatment abortions (n, %) | 60 | 41% |  | 47 | 35% |  | 24 | 45% |  |
| Total number of treatment abortions (n, %) | 88 | 18% |  | 78 | 14% |  | 49 | 21% |  |

**Supplementary Table 3**. SOFA Score-Standardized Mortality Ratio (SMR), Absolute Risk Reduction (AAR) and Number Needed to Treat (NNT) 28 days after the first ADVOS session and at the end of the registry in each of the subgroups. Only patients with a documented SOFA Score immediately before the first ADVOS treatment have been included in the analysis.

|  | **ALL (n = 202)** | | | | **Acidosis (n = 105)** | | | | **No Acidosis (n = 97)** | | | | **ACLF 3 (n = 49)** | | | |
| --- | --- | --- | --- | --- | --- | --- | --- | --- | --- | --- | --- | --- | --- | --- | --- | --- |
|  | n | expected deaths | observed deaths | observed deaths 28 days | n | expected deaths | observed deaths | observed deaths 28 days | n | expected deaths | observed deaths | observed deaths 28 days | n | expected deaths | observed deaths | observed deaths 28 days |
| n | 202 | 169 | 134 | 119 | 105 | 89 | 80 | 75 | 97 | 80 | 54 | 44 | 49 | 44 | 35 | 33 |
| mortality rate |  | 84% | 66% | 59% |  | 85% | 76% | 71% |  | 82% | 56% | 45% |  | 90% | 71% | 67% |
| AAR |  |  | 17% | 25% |  |  | 9% | 13% |  |  | 27% | 37% |  |  | 18% | 22% |
| NNT |  |  | 5,8 | 4,0 |  |  | 11,7 | 7,5 |  |  | 3,7 | 2,7 |  |  | 5,4 | 4,5 |
| SMR |  |  | 0,79 | 0,70 |  |  | 0,90 | 0,84 |  |  | 0,68 | 0,55 |  |  | 0,80 | 0,75 |
| CI 95% up |  |  | 0,93 | 0,83 |  |  | 1,10 | 1,03 |  |  | 0,86 | 0,71 |  |  | 1,06 | 1,01 |
| CI 95% low |  |  | 0,66 | 0,58 |  |  | 0,70 | 0,65 |  |  | 0,49 | 0,39 |  |  | 0,53 | 0,49 |

**Supplementary Table 4**. Course of treatment performance parameters in the subgroup of patients with respiratory acidosis. Median (IQR). Respiratory acidosis is defined as a blood pH < 7.35 and either a serum bicarbonate > 30 and a pCO2 > 45, or a serum bicarbonate ≥ 21 and a pCO2 > 45 being this higher than that expected according to Winter’s formula (pCO2 = HCO3 x 1,5 + 8).

|  |  | **ALL (n = 282)** | | |
| --- | --- | --- | --- | --- |
| **Parameter** | **Timepoint** | **Median** | **IQR 25** | **IQR 75** |
| Mean arterial pressure (mmHg) | Hospital Admission | 78 | 68 | 89 |
|  | Baseline | 70 | 62 | 77 |
|  | Post 1st Treatment | 73 | 63 | 78 |
|  | After Last Treatment | 67 | 52 | 76 |
| Bilirubin total (mg/dL) | Hospital Admission | 3,4 | 0,9 | 12,7 |
|  | Baseline | 5,0 | 1,7 | 9,1 |
|  | Post 1st Treatment | 5,5 | 2,4 | 7,5 |
|  | After Last Treatment | 6,3 | 3,5 | 9,0 |
| Potassium (mmol/L) | Hospital Admission | 4,5 | 3,9 | 5,0 |
|  | Baseline | 4,5 | 4,0 | 5,1 |
|  | Post 1st Treatment | 4,4 | 4,1 | 4,8 |
|  | After Last Treatment | 4,6 | 4,1 | 5,2 |
| Sodium (mmol/L) | Hospital Admission | 137 | 134 | 140 |
|  | Baseline | 139 | 136 | 143 |
|  | Post 1st Treatment | 140 | 137 | 142 |
|  | After Last Treatment | 140 | 138 | 144 |
| Creatinine (mg/dL) | Hospital Admission | 2,3 | 1,5 | 4,2 |
|  | Baseline | 2,1 | 1,4 | 3,3 |
|  | Post 1st Treatment | 1,6 | 1,1 | 2,5 |
|  | After Last Treatment | 1,4 | 0,8 | 1,9 |
| BUN (mg/dL) | Hospital Admission | 38 | 25 | 58 |
|  | Baseline | 32 | 22 | 50 |
|  | Post 1st Treatment | 18 | 13 | 34 |
|  | After Last Treatment | 15 | 9 | 31 |
| Chloride (mmol/L) | Hospital Admission | 105 | 97 | 107 |
|  | Baseline | 105 | 98 | 108 |
|  | Post 1st Treatment | 100 | 96 | 103 |
|  | After Last Treatment | 101 | 97 | 105 |
| Erythrocytes / RBC (10^12^/L) | Hospital Admission | 3,1 | 2,7 | 3,8 |
|  | Baseline | 2,8 | 2,4 | 3,2 |
|  | Post 1st Treatment | 2,5 | 2,2 | 3,0 |
|  | After Last Treatment | 2,5 | 2,3 | 3,1 |
| Leucocytes / WBC (10^9^/L) | Hospital Admission | 10,6 | 6,3 | 17,6 |
|  | Baseline | 11,8 | 7,7 | 21,8 |
|  | Post 1st Treatment | 16,0 | 9,5 | 19,9 |
|  | After Last Treatment | 16 | 12 | 27 |
| Platelet Count (10^9^/L) | Hospital Admission | 151 | 59 | 272 |
|  | Baseline | 91 | 50 | 226 |
|  | Post 1st Treatment | 58 | 33 | 153 |
|  | After Last Treatment | 47 | 28 | 123 |
| pH | Hospital Admission | 7,32 | 7,28 | 7,38 |
|  | Baseline | 7,22 | 7,11 | 7,30 |
|  | Post 1st Treatment | 7,37 | 7,31 | 7,45 |
|  | After Last Treatment | 7,39 | 7,29 | 7,43 |
| HCO3 (serum bicarbonate) (mmol/L) | Hospital Admission | 20,8 | 17,9 | 23,0 |
|  | Baseline | 21,3 | 17,9 | 23,4 |
|  | Post 1st Treatment | 26,0 | 21,7 | 30,6 |
|  | After Last Treatment | 24,9 | 18,9 | 36,3 |
| pCO_2_ (mmHg) | Hospital Admission | 46 | 33 | 58 |
|  | Baseline | 55 | 48 | 62 |
|  | Post 1st Treatment | 49 | 39 | 59 |
|  | After Last Treatment | 46 | 33 | 57 |
| Base Excess (mmol/L) | Hospital Admission | -4,6 | -7,8 | -0,5 |
|  | Baseline | -3,9 | -8,1 | -1,4 |
|  | Post 1st Treatment | 1,9 | -3,4 | 8,8 |
|  | After Last Treatment | 0,5 | -7,3 | 14,2 |
| Noradrenaline highest dose (µg/kg/min) | Hospital Admission | 0,613 | 0,370 | 0,815 |
|  | Baseline | 0,600 | 0,385 | 0,953 |
|  | Post 1st Treatment | 0,444 | 0,237 | 0,820 |
|  | After Last Treatment | 0,327 | 0,099 | 0,643 |
| Albumin (g/L) | Hospital Admission | 23 | 18 | 27 |
|  | Baseline | 22 | 18 | 27 |
|  | Post 1st Treatment | 21 | 16 | 27 |
|  | After Last Treatment | 18 | 12 | 20 |
| Lactate (mmol/L) | Hospital Admission | 2,9 | 1,5 | 7,9 |
|  | Baseline | 4,9 | 1,8 | 11,0 |
|  | Post 1st Treatment | 5,0 | 1,8 | 11,6 |
|  | After Last Treatment | 6,5 | 1,5 | 17,0 |
| Acidosis (n, % < pH 7.35) | Hospital Admission | 1 | 100% | 1,0 |
|  | Baseline | 1 | 100% | 1,0 |
|  | Post 1st Treatment | 1 | 100% | 1,0 |
|  | After Last treatment | 1 | 100% | 1,0 |

**Supplementary Table 5**. Paired differences of performance parameters between baseline and after the first treatment in the subgroup of patients with respiratory acidosis. Patients dead without data recording after the first ADVOS treatment session are excluded from the analysis since no pairing was possible. Respiratory acidosis is defined as a blood pH < 7.35 and either a serum bicarbonate > 30 and a pCO2 > 45, or a serum bicarbonate ≥ 21 and a pCO2 > 45 being this higher than that expected according to Winter’s formula (pCO2 = HCO3 x 1,5 + 8).

|  |  | **ALL (n = 282)** | | | |
| --- | --- | --- | --- | --- | --- |
| **Parameter** | **Pair** | **Mean** | **Lower** | **Upper** | **Sig.** |
| Mean arterial pressure | Baseline vs. post 1st treatment | -1,9 | -7,8 | 4,0 | 0,508 |
| Bilirubin total | Baseline vs. post 1st treatment | -1,5 | -3,2 | 0,3 | 0,093 |
| Potassium | Baseline vs. post 1st treatment | -0,1 | -0,4 | 0,1 | 0,280 |
| Sodium | Baseline vs. post 1st treatment | -0,8 | -3,0 | 1,5 | 0,494 |
| Creatinine | Baseline vs. post 1st treatment | -1,0 | -1,5 | -0,5 | **0,000** |
| BUN | Baseline vs. post 1st treatment | -12,1 | -17,8 | -6,3 | **0,000** |
| Chloride | Baseline vs. post 1st treatment | -2,3 | -7,2 | 2,5 | 0,330 |
| Erythrocytes / RBC | Baseline vs. post 1st treatment | -0,2 | -0,3 | 0,0 | **0,015** |
| Leucocytes / WBC | Baseline vs. post 1st treatment | 0,6 | -4,0 | 5,1 | 0,803 |
| Platelet Count | Baseline vs. post 1st treatment | -36,2 | -69,3 | -3,2 | **0,033** |
| PH | Baseline vs. post 1st treatment | 0,13 | 0,09 | 0,18 | **0,000** |
| HCO3 (serum bicarbonate) | Baseline vs. post 1st treatment | 6,5 | 3,8 | 9,1 | **0,000** |
| PCO2 | Baseline vs. post 1st treatment | -5,1 | -10,0 | -0,2 | **0,043** |
| Base Excess | Baseline vs. post 1st treatment | 7,9 | 4,6 | 11,2 | **0,000** |
| Noradrenaline highest dose | Baseline vs. post 1st treatment | -0,108 | -0,344 | 0,127 | 0,351 |
| Albumin | Baseline vs. post 1st treatment | -0,1 | -3,8 | 3,6 | 0,946 |
| Lactate | Baseline vs. post 1st treatment | -0,5 | -2,9 | 1,9 | 0,655 |
